# miR-34a regulates phenotypic modulation of vascular smooth muscle cells in intracranial aneurysm by targeting CXCR3 and MMP-2

**DOI:** 10.1590/1678-4685-GMB-2020-0124

**Published:** 2021-04-23

**Authors:** Xuesong Yuan, Xiaoxing Bian, Wenfeng Wei, Qing Bao, Ping Liu, Wenqing Jiang

**Affiliations:** 1 The Affiliated Hospital of Jiangsu University, Changzhou Wujin People’s Hospital, Department of Neurosurgery, Changzhou, China.

**Keywords:** miR-34a, CXCR3, MMP-2, intracranial aneurysms, vascular smooth muscle cell

## Abstract

MicroRNAs (miRNAs) dysregulation is tightly related to diseases including tumor, neuro disease and cardiovascular disease. In this study, we investigated the potential biological effects of miR-34a and its target CXCR3 in phenotypic modulation of vascular smooth muscle cells (VSMCs) of intracranial aneurysms (IAs). MiR-34a was found to be down-regulated in IAs patients tested by Real-time PCR and decreased in GEO data. Meanwhile, our study also showed miR-34a inhibited matrix metalloproteinases (MMPs) and migration of VSMCs. Besides, CXCR3 is a direct target of miR-34a identified via luciferase assay. CXCR3 showed inhibitory effect on SM-MHC, SM22 while promoted MMPs expression, cell proliferation and migration in VSMCs. MiR-34a reversed the effect of CXCR3 in VSMCs. In addition, MMP-2 is a competitive endogenous RNA (ceRNA) of CXCR3 sharing common miR-34a target. CXCR3 increased MMP-2 level through competitive endogenous RNA regulation by sponging endogenous miR-34a. In conclusion, miR-34a is down-regulated in IAs while CXCR3 is the direct target of miR-34a that regulates phenotypic modulation of VSMCs. CXCR3 increased MMP-2 level through competitive endogenous RNA regulation by sharing common miR-34a targets.

## Introduction

Intracranial aneurysms (IAs) are diseases with high mortality and morbidity rates, which are commonly caused by subarachnoid hemorrhage (SAH) (Bjorkman *et al*., 2017). The prevalence rate of IAs is about 2-3% in the general population all around the world (Kocur *et al*., 2018). As suggested by histological studies, cerebral aneurysms present with loss of internal elastic lamina of vascular wall; meanwhile, the loss of elastic fibers also enhances the outpouching of remaining vascular layers (Hamada *et al*., 2000). At the current stage, most unruptured IAs are discovered incidentally during the diagnostic imaging examination for common symptoms such as headaches. At present, surgical clipping and endovascular therapy are the major treatments for IAs, but these invasive treatments also give rise to potential serious complications that may decrease the patient life quality after surgery (Nam *et al*., 2019). It is urgently needed to investigate the pathology and molecular mechanisms of human IAs, so as to provide a promising non-invasive method for IAs and to prevent IAs rupture. Vascular smooth muscle cells (VSMCs) play vital roles in the phenotypic changes of IAs. During the change process, the contractile proteins SM-MHC and SM22 are down-regulated, and VSMCs show enhanced dissociation and migration abilities, which have been characterized as the pivotal pathogenesis of IAs (Belo *et al*., 2015; Ni *et al*., 2019; Yao *et al*., 2015). This study aimed to explore the detailed molecular mechanisms of IAs based on VSMCs, so as to provide a promising idea for the development of clinical treatment.

MicroRNAs (miRNAs) are a type of non-coding RNAs with the length of about 18-23 nucleotides (nt). MiRNAs can negatively regulate their target genes via recognizing the miRNA binding sites in the 3’-untranlated regions (3’UTR) of mRNAs (Yan *et al*., 2019). miRNA dysregulation is tightly related to diseases such as tumor, nerve disease and cardiovascular disease (CVD). In recent years, accumulating evidence has proved that miRNAs also play important roles in atherosclerosis (AS) and vascular remodeling (Chistiakov *et al*., 2015; Fang and Yeh, 2015; Petrica *et al*., 2020; Yao *et al*., 2015; Yu *et al*., 2020). Therefore, miRNAs may be the meaningful molecules related to the molecular mechanisms of IAs.

In different cancer types, C-X-C motif chemokine receptors (CXCRs) are connected with matrix metalloproteinases (MMPs) that promote cancer cell proliferation and migration (van der Meulen *et al*., 2009; Wang *et al*., 2014; Xia *et al*., 2018). This study investigated the concrete mechanisms involving miRNAs and CXCR3 and discovered that miR-34a, the well-known tumor suppressor miRNA, inhibited CXCR3 expression through regulating the phenotypic modulation of VSMCs in IAs.

## Subjects and methods

### Patients and samples

We recruited 20 patients who were diagnosed with IAs from the Department of Neurosurgery, Changzhou Wujin People’s Hospital during 01.2017 to 06.2019. Aneurysms size ranged from 21 × 16 mm to 2.4 × 1.1 mm. Meanwhile, the same amount of healthy subjects without any family history were employed as negative control group. The ages of patients did not show significant correlations to the size of aneurysms in both IAs group and control group (data not shown, P > 0.05). serum samples of all groups were obtained through centrifuging blood samples taken from subjects at 3500 × *g* for 10 min in room temperature, and then all serum samples were stored at -80 °C before experiments. IA tissues obtained through clipping surgery and removing from the site. Before using, all tissues were frozen in liquid nitrogen. Normal superficial temporal arteries were obtained from trauma patients with craniotomy treatments. All procedures were referred to previously (Xu *et al*., 2018). The study was approved by the Ethics Committee of Changzhou Wujin People’s Hospital (WJRMYY2017-38) and the study was performed in accordance with the ethical standards as laid down in the 1964 Declaration of Helsinki and its later amendments or comparable ethical standards. Informed consent was obtained from all subjects.

### Overexpression vector and reporter plasmid construction

CXCR3 3’UTR overexpression vector with miR-34a binding site was constructed. The primers used for PCR and vector construction were listed in Table 1.

**Table 1 -:** Primers were used in CXCR3 and MMP-2 3’UTR overexpression vector construction.

Genes	Forward primers 5’ to 3’	Reverse Primers 5’ to 3’
CXCR3 3’UTR	GGCCGGAATCCGGGCTCC	CAATAAACAAGATCGTCAG
MMP-2 3’UTR	GCTGGCCCTGGCTCCCACA	TCATCAATAAGATTCAACTA

For CXCR3 3’UTR overexpression vector, pcDNA3.1 (+) plasmid was used together with PCR product after sequencing. CXCR3 and MMP-2 3’UTR wild type and mutant type reporter plasmids were constructed.

### Cell culture and transfection

Human vascular smooth muscle cells (VSMCs; ATCC, USA) were cultured in Dulbecco’s Modified Eagle Medium (DMEM) with 10% FBS and incubated in 5% CO_2_ at 37 ℃. miR-34a mimics/inhibitor or negative control were transfected into VSMCs after seeding 24 h in 6-well plate. The concentration of all RNAs used for transfection was 50 nM based on the protocol of Lipofectamine 2000 (Invitrogen, USA). For CXCR4 overexpression plasmid transfection, the concentration of plasmid was 2 μg/well in a 6-well plate after 24 h of seeding. All analysis were conducted after 48 h transfection.

### Real-time PCR analysis (RT-PCR)

Cell total RNAs were extracted after different treatment. one milliliter of Trizol reagent (Invitrogen, USA) was used for each well in 6-well plate and extraction was conducted based on standard RNA extraction protocol. Real time PCR was conducted using cDNA template reversed transcribed from extracted RNAs. All results were normalized to GAPDH and calculated with the method of 2^-ΔΔCT^. The primers used are listed in Table 2.

**Table 2 -:** Primers were used in q-PCR analysis.

Genes	Forward primers 5’ to 3’	Reverse Primers 5’ to 3’
SM-MHC	GCTGCAGGTGACACGG	ATGATCAGAACAATAAAC
SM22	TCCTGTCTGTCCGAACCCA	GGGGAAAGAAGGCTTCCTCAG
MMP-2	TACAGGATCATTGGCTACACACC	GGTCACATCGCTCCAGACT
MMP-3	AGTCTTCCAATCCTACTGTTGCT	TCCCCGTCACCTCCAATCC
MMP-9	TGTACCGCTATGGTTACACTCG	GGCAGGGACAGTTGCTTCT
CXCR3	CCACCTAGCTGTAGCAGACAC	AGGGCTCCTGCGTAGAAGTT
CXCR4	ACTACACCGAGGAAATGGGCT	CCCACAATGCCAGTTAAGAAGA
CXCR6	GACTATGGGTTCAGCAGTTTCA	GGCTCTGCAACTTATGGTAGAAG

### Transwell assay

As for measuring migration ability of VSMCs, 10 ng/ml Mitomycin C was used and treated cells for 2 h. Then 1x10^5^ VSMCs in 200 μl medium without FBS were seeded into upper layer of the chamber (8 μm; BD Biosciences, USA). DMEM with 10% FBS were put into the lower layer of the chamber. After 24 h of seeding, violet crystalline solution was used to stain the cells attached on the lower layer of the chamber. Five random fields were chosen for each well to take pictures.

### Western blot

Total protein was extracted from cells using RIPA buffer. Protein was quantified by BCA method. For western blot, total 20 μg sample was loaded on the gel. After electrophoresis, PVDF membranes were used for protein transfer from the gel. Membranes were incubated with anti-MMP2 RabMAb (1:1000, Abcam, USA), anti-MMP3 (1:1000, Abcam, USA), anti-MMP9 (1:1000, Abcam, USA), anti-CXCR3 (1:1000, Abcam, USA), anti-CXCR4 (1:1000, Abcam, USA), anti-CXCR6 (1:1000, Abcam, USA) over night at 4 °C. All bands were normalized to anti-human β-actin monoclonal antibody (1:500, ZSGB-bio, China). ECL system (Thermo Fisher, USA) was used for imaging after incubating with secondary antibody.

### Luciferase assay

HEK 293T cells were co-transfected with miR-34a mimics and different luciferase reporter vectors. After 48 h transfection, luciferase activities were measured by microplate reader, and β-gal was added as internal reference.

### CCK-8 assay

VSMCs were seeded into 96-well plate when transfected with miR-34a and CXCR3 separately or combined at the density of 2 × 10^3^ cells/well. After incubation for different time points, microplate reader was used to measure the absorption of cells with different treatment at 450 nm with CCK-8 kit (Dojindo Laboratories, Japan). For each experiment, three isolated replications were conducted.

### RNA immunoprecipitation (RIP) assay

RNA immunoprecipitation was performed based on previously described. AGO2 (anti-AGO2, 1:50, Cell Signaling, USA) antibody was used and immunoprecipitated by 25 μl protein A/G agarose. Trizol agent was added to extract RNAs from the precipitant. And then, miR-34a, MMP2 and CXCR3 were measured by qPCR.

### Statistical analysis

Mean ± SD (standard deviation) was used for data calculation and presentation. All experiments were conducted for three times independently. The differences between the groups in patients were calculated by Mann Whitney U test performed with SPSS 22.0. Other comparisons between two groups were performed by using Student’s *t* tests. One-way analysis of variance (ANOVA) was used for comparisons more than two groups and performed by GraphPad 8.0. Differences with *p* values of less than 0.05 (*p) and 0.01 (**p) were considered as significant.

## Results

### miR-34a expression was down-regulated in IAs patients

**Figure 1 -:**
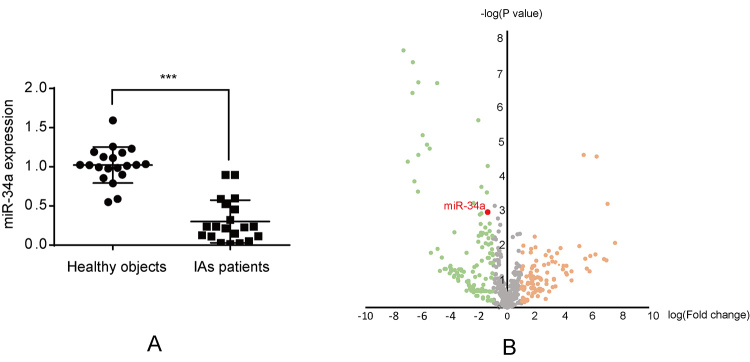
miR-34a expression. (A) Real-time PCR test for miR-34a in IA patients and healthy group, ***p < 0.0001. (B) GEO data (GSE46336) was downloaded from GEO datasets in NCBI and analyzed, in which, x axis represents log (fold change) and y axis represents log (-P value).

To identify the expression profile of miR-34a in IAs patients, a total of 20 IAs patients were recruited from the Department of Neurosurgery, Changzhou Wujin People’s Hospital, from January 2017 to June 2019. Additionally, 20 healthy subjects without any family history of IAs were recruited as controls. Real-time PCR (RT-PCR) was conducted to detect the serum miR-34a level. As shown in Figure 1A, miR-34a was significantly down-regulated in IAs patients compared with the control group. In addition, a GEO dataset (GSE46336) was analyzed. Similarly, miR-34a was found to be down-regulated in IAs tissues with the log [fold change, FC] value of -1.38, indicating that the miR-34a expression level in IAs tissues was only 4.17% compared with that in normal arterial tissues (Figure 1B).

### The role of miR-34a in the phenotypic modulation of VSMCs

**Figure 2 -:**
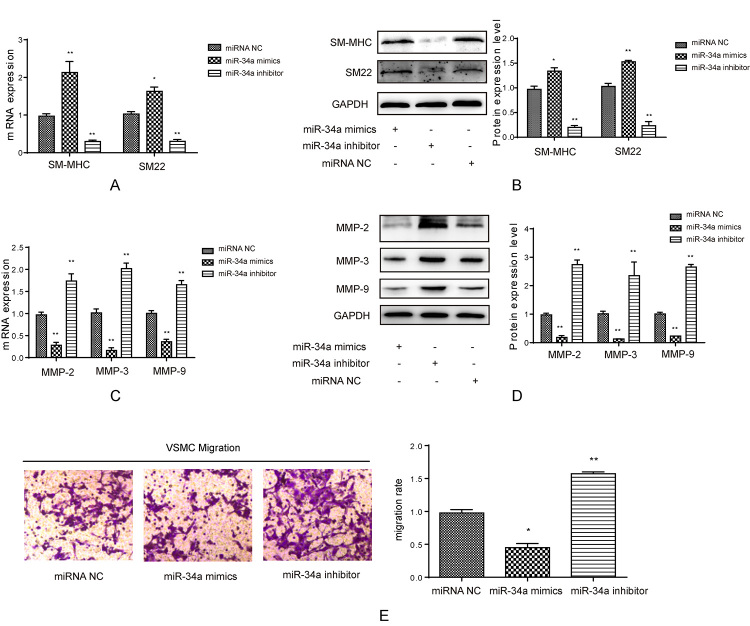
The effect of miR-34a on contractile proteins. (A) Real-time PCR was used to test SM-MHC and SM22 mRNA level when transfected with miR-34a mimics and inhibitor in smooth muscle cells, *p < 0.05, **p < 0.01, compared to miRNA NC group. (B) Western blot was used to test SM-MHC and SM22 protein level when transfected with miR-34a mimics and inhibitor in smooth muscle cells, quantification was calculated based on light density of bands in Quantity One, **p < 0.01 compared to miRNA NC group. (C) Real-time PCR was used to test MMP-2, MMP-3 and MMP-9 mRNA level when transfected with miR-34a mimics and inhibitor in smooth muscle cells, **p < 0.01 compared to miRNA NC group. (D) Western blot was used to test SM-MHC and SM22 protein level when transfected with miR-34a mimics and inhibitor in smooth muscle cells, quantification was calculated based on light density of bands in Quantity One, **p < 0.01 compared to miRNA NC group. (E) Transwell assay was used to detect migration ability of smooth muscle cells when treated with miR-34a mimcs/inhibitor, quantification was calculated based on OD value in each group and normalized to miRNA NC group, *p < 0.05, **p < 0.01 compared to miRNA NC group.

To understand the effect of miR-34a in IAs, both miR-34a mimics and inhibitor were transfected into VSMCs. After transfection, miR-34a mimics induced the mRNA and protein expression of smooth muscle myosin heavy chain (SM-MHC) and SM22, whereas miR-34a inhibitor suppressed their expression compared with control group (Figure 2A and 2B). The mRNA and protein expression levels of MMPs including MMP-2, MMP-3 and MMP-9 were inhibited by miR-34a mimic compared with control group (Figure 2C and 2D). In addition, to further examine the effect of miR-34a on the phenotypic modulation of VSMCs, VSMCs were transfected with miR-34a mimics/inhibitor to detect their migration rate. It was shown in Figure 2E that miR-34a mimics significantly inhibited the migration of VSMCs, while miR-34a inhibitor induced their migration. Thus, miR-34a changed the phenotypic modulation and inhibited the migration of VSMCs.

### Several cytokine receptors and chemokine genes were up-regulated in IAs patients

**Figure 3 -:**
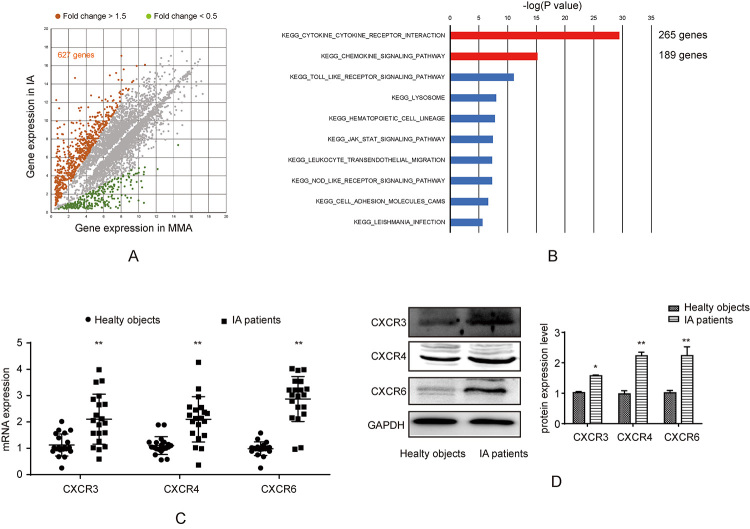
CXCRs expression in IAs. (A) GEO data (GSE46338) was downloaded from GEO datasets in NCBI and analyzed, in which, x axis represents gene expression in MMA and y axis represents log gene expression in IAs. The dots of genes with fold change over 1.5 were marked brown (627 genes were found). (B) KEGG signaling analysis of 627 genes obtained from Figure 3A. (C) Real-time PCR was used to test CXCR3, CXCR4 and CXCR6 in tissues from IAs patients and healthy subjects group, **p < 0.01. (D) Western blot was used to test CXCR3, CXCR4 and CXCR6 in tissues from IAs patients and control group, quantification was calculated based on light density of bands and normalized to GAPDH, *p < 0.05, **p < 0.01 compared to healthy objects group.

To obtain the potential dysregulated (especially up-regulated) genes in IAs patients, the GSE data (GSE 46338) was downloaded and analyzed. Compared with normal arterial tissues, there were altogether 627 genes with FC>1.5 (Figure 3A). Thereafter, all these 627 genes were incorporated into signaling pathway analysis against the KEGG database. As a result, the cytokine receptors and chemokine genes were the most significantly enriched (Figure 3B). In addition, accumulating studies have suggested that CXCRs are involved in cell migration, which is an important consequence of the phenotypic modulation of VSMCs in IAs. In this study, the expression levels of CXCR3, CXCR4 and CXCR6 were checked by RT-PCR IAs tissues (n = 20). Compared with normal tissues (n = 20), the expression levels of these three CXCRs were remarkably up-regulated within IAs tissues (Figure 3C). Western blotting analysis suggested that, CXCR3, CXCR4 and CXCR6 were up-regulated within IAs tissues, which was similar to RT-PCR results (Figure 3D).

### CXCR3 was a direct target of miR-34a

**Figure 4 -:**
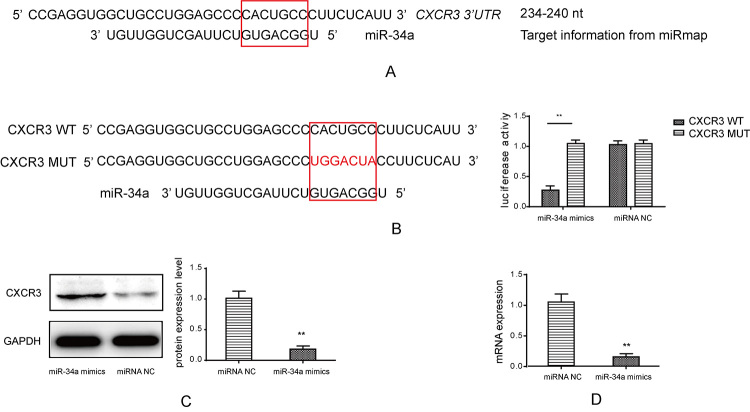
Target confirmation between CXCR3 and miR-34a. (A) The software miRmap was used to predict the target between CXCR3 and miR-34a. (B) Luciferase assay was used to identify direct target between CXCR3 and miR-34a, the target sequence was mutated and cloned into luciferase reporter plasmid, the luciferase activity decreased in CXCR3 WT group, **p < 0.01 compared to CXCR3 MUT group. (C) Western blot was used to detect CXCR3 protein level with miR-34a mimics transfection, quantification was calculated based on light density of bands, miR34a mimics reduced protein level compared to miRNA NC, **p < 0.01. (D) Real-time PCR was used to test CXCR3 with miR-34a mimics transfection, miR-34a inhibited mRNA expression of CXCR3 compared to miRNA NC treatment, **p < 0.01.

CXCRs were up-regulated within IAs tissues while miR-34a was down-regulated. Therefore, we further analyzed the potential target relations between miR-34a and CXCRs based on the miRNA regulation principles. As a result, although CXCR3, CXCR4 and CXCR6 were up-regulated in IAs tissues, only CXCR3 was identified as a potential target of miR34a with binding site on its 3’UTR (Figure 4A). Indeed, in luciferase assay, reporter plasmid with CXCR3 wild type (CXCR3 WT) sequence that contained the specific binding site showed decreased luciferase activity when it was co-transfected with miR-34a mimics in HEK293 cells, but such a result was not observed in the reporter plasmid with the CXCR3 mutant type (CXCR3 MUT) sequence (Figure 4B). In addition, when VSMCs were transfected with miR-34a mimics, the basal CXCR3 mRNA and protein levels were significantly inhibited by miR-34a compared with those in miRNA NC (Figure 4C and 4D). The above results indicated that CXCR3 was a direct target of miR-34a.

### miR-34a reversed the effect of CXCR3 on the proliferation, migration and phenotypic modulation of VSMCs

**Figure 5 -:**
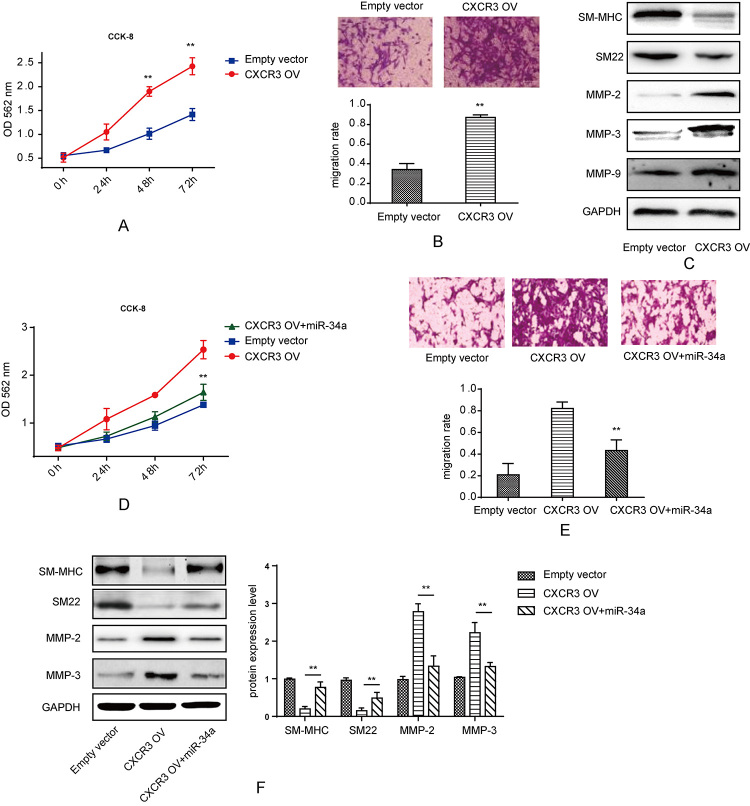
The effect of CXCR3 on cell proliferation and contractile proteins expression. (A) CCK-8 kit was used to detect proliferation of VSMCs when transfected with CXCR3 OV and empty vector, CXCR3 overexpression induced proliferation compared to empty vector group, **p < 0.01. (B) Transwell assay was used to detect migration ability of smooth muscle cells when treated with CXCR3 OV and empty vector, quantification was calculated based on OD value in each group and normalized to empty vector group, *p < 0.05, **p < 0.01. (C) Western blot was used to test SM-MHC, SM22, MMP-2, MMP-3 and MMP-9 protein level when transfected with CXCR3 OV and empty vector in smooth muscle cells. (D) CCK-8 kit was used to detect proliferation of VSMCs when transfected with CXCR3 OV and empty vector or co-transfected with miR-34a mimics, co-transfection of miR-34a and CXCR3 OV reversed the proliferation rate of VSMCs compared to CXCR3 OV group, **p < 0.01. (E) Transwell assay was used to detect migration ability of smooth muscle cells when treated with CXCR3 OV and empty vector or co-transfected with miR-34a, quantification was calculated based on OD value in each group and normalized to empty vector group, co-transfection of miR-34a and CXCR3 OV reversed the migration rate of VSMCs compared to CXCR3 OV group **p < 0.01. (F) Western blot was used to test SM-MHC, SM22, MMP-2, MMP-3 protein level when transfected with CXCR3 OV and empty vector or co-transfected with miR-34a mimics in smooth muscle cells, quantification was calculated based on light density of bands in Quantity One, **p < 0.01.

CXCR3 was up-regulated in IAs patients. Consequently, to understand the detailed role of CXCR3 in VSMCs, we designed the CXCR3 over-expression vector (CXCR3 OV). After transfection with CXCR3 OV, the proliferation rate increased within 72 h, as tested by CCK-8 assay (Figure 5A). With regard to migration, VSMCs showed a higher migration rate by CXCR3 overexpression (Figure 5B). Results of western blotting analysis suggested that, the expression of SM-MHC and SM22 decreased while that of MMP-2 and MMP-3 increased by CXCR3 overexpression (Figure 5C). According to the direct target relation between miR-34a and CXCR3, we tested the effect of miR-34a on CXCR3 overexpression. Compared with CXCR3 overexpression alone, co-transfection of miR-34a mimics with CXCR3 OV showed a reversed effect on the proliferation of VSMCs (Figure 5D). miR-34a also exhibited a reversed effect on the migration ability VSMCs after co-transfection with CXCR3 OV (Figure 5E). In western blotting assay, miR-34a partially rescued the protein expression of SM-MHC and SM22 and inhibited the CXCR3-induced increase in MMP2 and MMP3 expression.

### MMP-2 was a ceRNA of CXCR3 that shared the common miR-34a target

**Figure 6 -:**
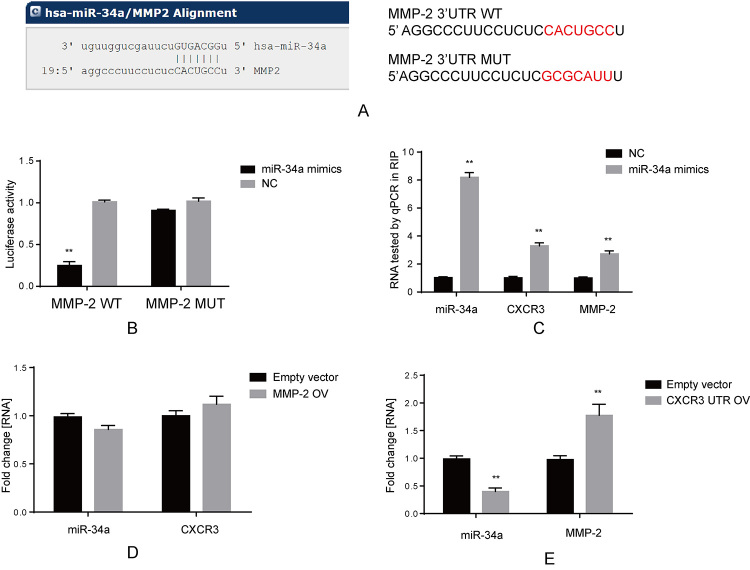
Interactions confirmed among CXCR3, miR-34a and MMP-2. (A) Online prediction software was used to find potential target between MMP-2 and miR-34a (left), and seed sequence was mutated in MMP-2 3’UTR (right). (B) Luciferase assay was used to identify direct target between MMP-2 and miR-34a, the target sequence was mutated and cloned into luciferase reporter plasmid, **p < 0.01. (C) RIP assay was used to detect binding among miR-34a, MMP-2 and CXCR3. All of these three RNAs were enriched when transfected with miR-34a mimics and compared to NC group, **p < 0.01, tested by q-PCR. (D) Overexpression MMP-2 did not change the level of miR-34a and CXCR3 in VSMCs. (E) Overexpression CXCR3 3’UTR suppressed miR-34a and increased MMP-2 in VSMCs, **p < 0.01, tested by q-PCR.

It was found that several MMPs were inhibited by miR-34a mimics, while the overexpression of CXCR3 reversed the effect of miR-34a on MMPs. Therefore, we further examined whether these MMPs were targeted by miR-34a and formed competitive endogenous RNA (ceRNA) regulation together with CXCR3. Among MMP-2, MMP-3 and MMP-9, only MMP-2 was predicted to contain the potential miR-34a target in its 3’UTR (Figure 6A). Next, this target was confirmed by luciferase assay. As shown in Figure 6B, after transfection with miR-34a mimics, only the reporter vector harboring MMP-2 3’UTR wild type sequence exhibited decreased luciferase activity. In addition, results of RNA binding immunoprecipitation assay suggested that, compared with NC, the 3’UTR of both CXCR3 and MMP-2 were enriched by immunoprecipitation via AGO2 antibody after transfection with miR-34a mimics (Figure 6C). To confirm whether MMP-2 induced CXCR3 expression, the overexpression vector containing MMP-2 3’UTR sequence (MMP-2 OV) was transfected into VSMCs. According to our results, no remarkable change was detected in miR-34a or CXCR3 (Figure 6D), whereas the overexpression vector containing CXCR3 3’UTR sequence (CXCR3 UTR OV) suppressed the expression of miR-34a and induced that of MMP-2 relative to empty vector (Figure 6E). Based on the results in Figure 5E and 5F, MMP-2 formed ceRNA regulation together with CXCR3 and miR-34a, and CXCR-3 was located at the upstream of this CXCR3/miR-34a/MMP-2 regulatory axis.

## Discussion

Based on the current clinical treatment and research, it is difficult to find an effective therapy for IAs apart from surgical procedure (Hamada *et al*., 2000). In addition, the resultant SAH after rupture is severe and has negative social impact, so it is necessary to investigate the mechanisms underlying IAs formation and rupture, and to develop novel therapies based on the identified molecular mechanisms. In the present study, we confirmed that the serum miR-34a level significantly decreased in IAs patients compared with controls, which suggested that miR-34a had a positive effect and was involved in IAs formation and progression.

As an miRNA member, prior studies have indicated that miR-34a is deregulated in various cancers (Li *et al*., 2019; Liu *et al*., 2020; Xiu *et al*., 2020). Besides, it is the first miRNA found to be directly regulated by p53 (Rokavec *et al*., 2014). Accumulating evidence reveals that miR-34a inhibits tumorigenesis through regulating epithelial mesenchymal transition (EMT). However, the effect of miR-34a on IAs has not been clarified yet. IAs are characterized by the loss of arterial wall integrity where extracellular matrix (ECM) and inflammation deregulation are found. In this process, VSMCs are vital for blood pressure regulation and vascular plasticity under certain disease conditions. In IAs, VSMCs are found to alter from contractile state to the matrix remodeling phenotype, and during the change process, VSMCs have promoted migration and proliferation abilities. Thus, it is interesting to investigate the effect and potential mechanism of miR-34a in IAs.

In this study, miR-34a was found to be down-regulated in IAs patients at our hospital, which indicated that it was involved in IAs progression. Actually, previous sequencing data obtained from the GEO database also show a low expression level of miR-34a in IAs tissues. Meanwhile, our study suggested that miR-34a inhibited MMPs and the migration of VSMCs. Due to the inducible role of inflammation in IAs, we further analyzed the gene expression profiles in IAs. Unsurprisingly, cytokine and chemokine genes were highly expressed in IAs. In different cancer types, CXCRs are connected with MMPs that promote cancer migration. CXCR3 plays an important role in angiogenesis, inflammation and cancer (Lacotte *et al*., 2009). In addition, LRP1 regulates CXCR3 to suppress the invasion of primary brain tumors. CXCR3 also promotes the migration of CD4+ T cells by ICP4 (Boye *et al*., 2017). Further, CXCR4 induces the migration of mesenchymal stem cells together with SDF-1α (Deng *et al*., 2018). In bone marrow derived cells, erythropoietin exhibits the protective effect by suppressing apoptosis via CXCR4 (Li *et al*., 2015). In addition to CXCR3 and CXCR4, other CXCRs are also involved in migration and inflammation. Thus, it is intriguing to make clear the functions of CXCRs in IAs. From online software, CXCR3 was identified as a direct target of miR-34a, as verified by luciferase assay. Furthermore, in functional analysis, CXCR3 showed inhibitory effect on SM-MHC and SM22 but promoted MMPs, proliferation and migration of VSMCs. CXCR3 was a direct target of miR-34a and miR-34a reversed the effect of CXCR3 on VSMCs, which revealed that miR-34a had a protective effect on IAs. In addition, MMP-2 was first identified as a direct target of miR-34a, which formd ceRNA regulation together with CXCR3 and miR-34a. Meanwhile, in this ceRNA regulation, CXCR3 functions as a sponge that absorbs miR-34a, which leads to the increased MMP-2 expression. In this study, we also tested the serum miR-34a expression in IAs patients, and discovered that the circulating miRNAs might play important roles in IAs. Indeed, various circulating miRNAs have been considered as the potential molecular biomarkers for IA rupture (Supriya *et al*.*,* 2020). Although the molecular mechanism of miR-34a involvement has been clarified in the cell model that is limited for testing the function of circulating miR-34a, it is still necessary to confirm the function and effect of circulating miR-34a in IAs mouse models.

In conclusion, miR-34a is down-regulated in IAs, while CXCRs including CXCR3, CXCR4 and CXCR6 are up-regulated. Among these CXCRs, CXCR3 is the direct target of miR-34a that regulates the phenotypic modulation of VSMCs in IAs by targeting CXCR3. CXCR3 increases the MMP-2 expression through ceRNA regulation by sharing the common miR-34a target.

## References

[B1] Belo VA, Guimaraes DA, Castro MM (2015). Matrix metalloproteinase 2 as a potential mediator of vascular
smooth muscle cell migration and chronic vascular remodeling in
hypertension. J Vasc Res.

[B2] Bjorkman J, Frosen J, Tahtinen O, Backes D, Huttunen T, Harju J, Huttunen J, Kurki MI, von Und Zu Fraunberg M, Koivisto T (2017). Irregular shape identifies ruptured intracranial aneurysm in
subarachnoid hemorrhage patients with multiple aneurysms. Stroke.

[B3] Boye K, Pujol N, I DA, Chen YP, Daubon T, Lee YZ, Dedieu S, Constantin M, Bello L, Rossi M (2017). The role of CXCR3/LRP1 cross-talk in the invasion of primary
brain tumors. Nat Commun.

[B4] Chistiakov DA, Sobenin IA, Orekhov AN, Bobryshev YV (2015). Human miR-221/222 in physiological and atherosclerotic vascular
remodeling. Biomed Res Int.

[B5] Deng QJ, Xu XF, Ren J (2018). Effects of sdf-1/cxcr4 on the repair of traumatic brain injury in
rats by mediating bone marrow derived mesenchymal stem cells. Cell Mol Neurobiol.

[B6] Fang YC, Yeh CH (2015). Role of microRNAs in vascular remodeling. Curr Mol Med.

[B7] Hamada J, Yano S, Kai Y, Koga K, Morioka M, Ishimaru Y, Ushio Y (2000). Histopathological study of venous aneurysms in patients with
dural arteriovenous fistulas. J Neurosurg.

[B8] Kocur D, Przybylko N, Bazowski P, Baron J (2018). Rupture during coiling of intracranial aneurysms: Predictors and
clinical outcome. Clin Neurol Neurosurg.

[B9] Lacotte S, Brun S, Muller S, Dumortier H (2009). CXCR3, inflammation, and autoimmune diseases. Ann N Y Acad Sci.

[B10] Li J, Guo W, Xiong M, Han H, Chen J, Mao D, Tang B, Yu H, Zeng Y (2015). Effect of SDF-1/CXCR4 axis on the migration of transplanted bone
mesenchymal stem cells mobilized by erythropoietin toward lesion sites
following spinal cord injury. Int J Mol Med.

[B11] Li Z, Chen H (2019). miR-34a inhibits proliferation, migration and invasion of
paediatric neuroblastoma cells via targeting HNF4alpha. Artif Cells Nanomed Biotechnol.

[B12] Liu F, Ai FY, Zhang DC, Tian L, Yang ZY, Liu SJ (2020). LncRNA NEAT1 knockdown attenuates autophagy to elevate 5-FU
sensitivity in colorectal cancer via targeting miR-34a. Cancer Med.

[B13] Nam JS, Jeon SB, Jo JY, Joung KW, Chin JH, Lee EH, Chung CH, Choi IC (2019). Perioperative rupture risk of unruptured intracranial aneurysms
in cardiovascular surgery. Brain.

[B14] Ni T, Gao F, Zhang J, Lin H, Luo H, Chi J, Guo H (2019). Impaired autophagy mediates hyperhomocysteinemia-induced HA-VSMC
phenotypic switching. J Mol Histol.

[B15] Petrica L, Pusztai AM, Vlad M, Vlad A, Gadalean F, Dumitrascu V, Vlad D, Velciov S, Gluhovschi C, Bob F (2020). MiRNA expression is associated with clinical variables related to
vascular remodeling in the kidney and the brain in type 2 diabetes mellitus
patients. Endocr Res.

[B16] Rokavec M, Li H, Jiang L, Hermeking H (2014). The p53/miR-34 axis in development and disease. J Mol Cell Biol.

[B17] Supriya M, Christopher R, Indira Devi B, Bhat DI, Shukla D (2020). Circulating microRNAs as potential molecular biomarkers for
intracranial aneurysmal rupture. Mol Diagn Ther.

[B18] van der Meulen AA, Biber K, Lukovac S, Balasubramaniyan V, den Dunnen WF, Boddeke HW, Mooij JJ (2009). The role of CXC chemokine ligand (CXCL)12-CXC chemokine receptor
(CXCR)4 signalling in the migration of neural stem cells towards a brain
tumour. Neuropathol Appl Neurobiol.

[B19] Wang Y, Fu W, Zhang S, He X, Liu Z, Gao D, Xu T (2014). CXCR-7 receptor promotes SDF-1alpha-induced migration of bone
marrow mesenchymal stem cells in the transient cerebral ischemia/reperfusion
rat hippocampus. Brain Res.

[B20] Xia R, Xu G, Huang Y, Sheng X, Xu X, Lu H (2018). Hesperidin suppresses the migration and invasion of non-small
cell lung cancer cells by inhibiting the SDF-1/CXCR-4
pathway. Life Sci.

[B21] Xiu C, Jiang J, Song R (2020). Expression of miR-34a in cataract rats and its related
mechanism. Exp Ther Med.

[B22] Xu J, Yan S, Tan H, Ma L, Feng H, Han H, Pan M, Yu L, Fang C (2018). The miR-143/145 cluster reverses the regulation effect of KLF5 in
smooth muscle cells with proliferation and contractility in intracranial
aneurysm. Gene.

[B23] Yan B, Wang H, Tan Y, Fu W (2019). microRNAs in cardiovascular disease: Small Molecules but Big
Roles. Curr Top Med Chem.

[B24] Yao Y, Norris EH, Strickland S (2015). The cellular origin of laminin determines its role in blood
pressure regulation. Cell Mol Life Sci.

[B25] Yu DR, Wang T, Huang J, Fang XY, Fan HF, Yi GH, Liu Q, Zhang Y, Zeng XZ, Liu QB (2020). MicroRNA-9 overexpression suppresses vulnerable atherosclerotic
plaque and enhances vascular remodeling through negative regulation of the
p38MAPK pathway via OLR1 in acute coronary syndrome. J Cell Biochem.

